# A Novel Cationic Niosome Formulation for Topical Delivery of Adapalene

**DOI:** 10.1155/bmri/8874333

**Published:** 2025-10-19

**Authors:** Hadis Tabatabaie-Mehr, Rassol Haddadi, Setareh Isfahani-Nia, Seyed Yaser Vafaei

**Affiliations:** ^1^ Department of Pharmaceutics, School of Pharmacy, Hamadan University of Medical Sciences, Hamadan, Iran, umsha.ac.ir; ^2^ Department of Pharmacology and Toxicology, School of Pharmacy, Hamadan University of Medical Sciences, Hamadan, Iran, umsha.ac.ir

**Keywords:** adapalene, dermal drug delivery, hydrogel, niosome

## Abstract

In this study, we developed and optimized an adapalene (Ada)‐loaded niosomal hydrogel using the response surface methodology (RSM) and evaluated its efficacy for acne treatment. Ada‐loaded niosomes (Ada‐Nio) were prepared using the thin‐film hydration (TFH) method, and their physicochemical characteristics, including particle size, zeta potential, entrapment efficiency (EE%), and morphology, were determined. After preparing the Ada‐Nio hydrogel, its viscosity, pH, in vitro release behavior, and cytotoxicity were evaluated in the human dermal fibroblast (HDF) cell line using the MTT assay. The Draize test was performed to evaluate skin irritation caused by the Ada‐Nio hydrogel in New Zealand rabbits. The particle size, zeta potential, polydispersity index (PDI), and EE% of the optimized Ada‐Nio formulation were 168.8 ± 1.5 nm, −70.6 ± 1.05 mV, 0.273 ± 0.015, and 80.3 ± 2.8*%*, respectively. SEM images of Ada‐Nio showed spherical morphology. The viscosity and pH of the Ada‐Nio hydrogel were 33,840 ± 1200 cP and 6.5 ± 0.3, respectively. The in vitro release profile showed that 73 ± 2.1*%* of Ada was released from the niosomal hydrogel within 24 h. Results from the cell viability and Draize tests indicated that the Ada‐Nio hydrogel can be considered a safe and effective dermal agent for acne treatment. Niosomal hydrogel formulations appear to be safe, effective, and promising nanocarriers for the dermal delivery of Ada.

## 1. Introduction

The term acne usually refers to acne vulgaris, which is the most common skin disorder in adolescents and young adults [1]. Acne is a chronic inflammatory dermatosis that is usually divided into two categories: noninflammatory (open and closed comedones) and inflammatory (papules and pustules) [2]. In acne, the proliferation and abnormal differentiation of keratinocytes increase sebum production. This sebum provides a favorable environment for the proliferation of a type of Gram‐positive bacteria called *Cutibacterium acnes*, which triggers an inflammatory response due to bacterial antigens and cytokines [3].

Although there is no definitive cure for acne vulgaris, topical and systemic treatments can reduce its severity and associated scarring [4]. All available drug treatments act through mechanisms that inhibit acne pathogenesis, including normalizing follicular keratinization, reducing sebum production, inhibiting *Cutibacterium acnes*, and suppressing inflammation [5].

As a vitamin A derivative, retinoids are used as first‐line therapy for both inflammatory and comedonal acne. Their mechanism of action involves preventing abnormal cell differentiation, reducing sebum production, and inhibiting microcomedone formation [6, 7].

Adapalene is a third‐generation retinoid with anti‐inflammatory and keratolytic effects used in the treatment of acne [8]. However, its physicochemical properties limit penetration into the skin and hair follicles, thereby reducing topical bioavailability [9]. Although adapalene has better tolerability than first‐generation retinoids, local side effects such as erythema, irritation, dryness, and photosensitivity have been reported [8].

In recent years, extensive research has focused on developing adapalene‐loaded nanocarriers—such as nanoemulsions, nanosuspensions, micelles, liposomes, polymeric nanoparticles, and solid lipid nanoparticles—to enhance topical bioavailability, enable controlled drug release, improve stability, and minimize side effects [10, 11].

Niosomes are vesicular carriers composed of nonionic surfactants and cholesterol or its derivatives [12]. Their unique structure allows encapsulation of both hydrophilic and lipophilic drugs: Hydrophilic compounds are entrapped in the aqueous core or adsorbed on the bilayer surface, whereas lipophilic compounds are incorporated within the bilayer membrane [13]. Niosomes have gained significant attention due to their enhanced stability, controlled drug release, high entrapment efficiency (EE%), biocompatibility, nontoxicity, and improved patient compliance [14, 15].

Hydrogels are polymeric materials with hydrophilic networks capable of retaining large amounts of water. Their swelling capacity and elasticity make them suitable for use as continuous drug delivery systems with controlled release profiles [16, 17].

Considering that niosomes enhance drug penetration into the skin and that hydrogels prolong drug residence time at the application site, we developed an adapalene‐loaded niosomal hydrogel as a potential drug delivery system for acne treatment. In this study, we utilized response surface methodology (RSM) to optimize and characterize adapalene‐loaded niosomes (Ada‐Nio) and formulated an Ada‐Nio hydrogel to evaluate its in vitro skin penetration and dermal safety.

## 2. Materials and Methods

### 2.1. Chemicals and Reagents

Adapalene was purchased from Darou Pakhsh Pharmaceutical Co. (Tehran, Iran). Cetyltrimethylammonium bromide (CTAB) and Span 60 were obtained from Fagron Co. (Shanghai, China) and Brasquim Co. (Brazil), respectively. Cholesterol, chloroform, dimethylformamide (DMF), ethanol, HPLC‐grade methanol, and acetonitrile were purchased from Samchun Pure Chemical Co. Ltd. (Seoul, South Korea), and Carbopol 934 (CBP 934) was obtained from Lubrizol Corp. (Wickliffe, Ohio, United States). Propylene glycol, glycerin, and triethanolamine (TEA) were purchased from Merck (Darmstadt, Germany). Analytical‐grade water was prepared using a Millipore purification system. All other chemicals were also purchased from Merck (Germany).

### 2.2. Ada‐Nio Preparation

The thin‐film hydration (TFH) method was used to prepare Ada‐Nio with minor modifications [18]. Adapalene, Span 60, CTAB, and cholesterol were dissolved in a solvent mixture of chloroform and methanol (2:1 v/v), then stirred and sonicated for 10 min. The resulting solution was transferred to a round‐bottom flask, and a thin film was formed on the inner wall using a rotary evaporator at 55°C for 60 min under vacuum. Based on previous studies, adapalene remains stable at this temperature [19–21].

Subsequently, 20 mL of phosphate buffer (pH 7.4) was added to the flask and sonicated for 20 min. The mixture was hydrated for 1 h at 52°C using a rotary device, then sonicated in an ice bath for 20 min. The resulting nanoparticles were stored at 4°C for further studies.

Fifteen formulations were designed by varying the amounts of adapalene and other components to optimize Ada‐Nio properties while keeping other parameters constant. Each formulation was prepared in triplicate using the same method. Table 1 summarizes the independent and dependent variables and their levels, while Table 2 details the sequence of the different Ada‐Nio formulations.

**Table 1:** Range of variables used in the central composite design.

	**Levels**	
Independent variables (factors)	−1	+1
A: Adapalene (mg)	10	20
B: CTAB (mg)	2.73	27.3
C: Span 60/cholesterol ratio	1	2
Dependent variables (responses)	Constrains	
*Y*_1_: Size (nm)	Minimize	
*Y*_2_: Zeta potential (mV)	Maximize	
*Y*_3_: EE (%)	Maximize	

**Table 2:** Variable amount of the Ada‐Nio formulation.

**Run**	**Adapalene (mg)**	**CTAB (mg)**	**Span 60/cholesterol ratio**
Ada‐Nio‐1	20	27.3	1.5
Ada‐Nio‐2	10	15.02	1
Ada‐Nio‐3	15	27.3	2
Ada‐Nio‐4	15	2.73	1
Ada‐Nio‐5	20	2.73	1.5
Ada‐Nio‐6	10	2.73	1.5
Ada‐Nio‐7	15	15.02	1.5
Ada‐Nio‐8	15	15.02	1.5
Ada‐Nio‐9	10	27.3	1.5
Ada‐Nio‐10	20	15.02	1
Ada‐Nio‐11	15	15.02	1.5
Ada‐Nio‐12	15	27.3	1
Ada‐Nio‐13	20	15.02	2
Ada‐Nio‐14	10	15.02	2
Ada‐Nio‐15	15	2.73	2

### 2.3. Particle Size and Zeta Potential

The particle size, polydispersity index (PDI), and zeta potential were measured by photon correlation spectroscopy (PCS) and laser Doppler velocimetry (LDV) using a Malvern Zetasizer Nano ZS (Malvern Instruments Ltd., Malvern, United Kingdom). Briefly, Ada‐Nio was diluted with double‐distilled water at a ratio of 1:20, followed by sonication for 5 min in an ultrasonic bath (Bandelin SONOREX, Berlin, Germany). All measurements were performed in triplicate at 25°C.

### 2.4. Adapalene EE%

The EE% of adapalene in niosomes was determined as follows: Freshly prepared nanoparticles were diluted with ethanol (1:4) and centrifuged in an ultracentrifuge (Beckman Coulter, United States) at 9000 rpm for 25 min at 25°C. The sediment was collected, and the supernatant was analyzed for free adapalene content by HPLC [22]. The HPLC system (Shimadzu, Japan) consisted of an LC‐20ADXR pump and PDA/SPD‐M20A detector. The mobile phase was a mixture of water and acetonitrile (10:90, v/v), adjusted to pH≈3 using concentrated phosphoric acid, and delivered at a flow rate of 1.3 mL/min through a C18 column (4.6 × 250 mm, ODS‐3 Perfectsil Target; MZ Company, Germany). The injection volume was 20 *μ*L, and detection was performed at 235 nm. A standard calibration curve of adapalene was linear in the range of 10–100 *μ*g/mL (*R* ^2^ = 0.9994).

The EE% was calculated as follows:

EE%=total drug content−free drug total drug content∗100

### 2.5. Morphology

The surface morphology of the optimized formulation was characterized by scanning electron microscopy (SEM), as previously described [22, 23]. Briefly, a small amount of nanoparticle suspension was diluted, deposited as a thin layer on an aluminum stub, and dried in a desiccator at 25°C for 8 h. The dried samples were coated with a thin layer of gold under vacuum using a Desk V Denton sputter coater (Moorestown, United States) to ensure conductivity. SEM images were obtained at an accelerating voltage of 20 kV at suitable magnifications [24].

### 2.6. Experimental Design and Optimization

A central composite design (CCD) was employed to statistically optimize the Ada‐Nio formulation using Design‐Expert software (V. 7.0.0, Stat‐Ease Inc., Minneapolis, United States). Three independent variables (factors) were considered: adapalene concentration (milligram) (A), CTAB amount (milligram) (B), and Span 60/cholesterol ratio (C). The responses evaluated were particle size (*Y*_1_), zeta potential (*Y*_2_), and EE% (*Y*_3_). The factor ranges were determined from preliminary experiments and are summarized in Table 1. Based on the software’s design matrix, 15 experimental batches (Ada‐Nio‐1 to Ada‐Nio‐15) were prepared in triplicate, as listed in Table 2.

### 2.7. Cell Culture and MTT Assay

The cytotoxicity of adapalene, Ada‐Nio, and blank niosomes was evaluated using the MTT assay. Human dermal fibroblast (HDF) cells were seeded in 96‐well plates at 1.0 × 10^4^ cells/well and incubated for 48 h to reach 80%–85% confluence. Cells were then treated with five concentrations (0, 1, 10, 50, and 100 *μ*g/mL) of each formulation. After 24 h exposure, MTT solution (5 mg/mL) was added and incubated at 37°C for 4 h. Formazan crystals were dissolved in 150 *μ*L of DMSO per well, and absorbance was measured at 570 nm using a Synergy HTX microplate reader (Germany). Cell viability was calculated as the percentage of treated cells relative to untreated controls (100%).

### 2.8. Adapalene‐Loaded Niosomal Hydrogel Preparation

To prepare a hydrogel from the optimized Ada‐Nio suspension, methylparaben (0.2% w/w) and propylparaben (0.02% w/w) were dissolved in 5 mL of propylene glycol (5% v/v). Then, glycerin (1% w/v) was added to 5 mL of deionized water, and the two solutions were combined under stirring. CBP 934 powder (0.4% w/w) was added gradually to obtain a uniform solution. The Ada‐Nio suspension was then added dropwise and homogenized for 10 min using a propeller mixer to ensure uniformity. Finally, a few drops of TEA were added to form a hydrogel and adjust the pH to 6.2. A blank niosomal hydrogel and a hydrogel containing standard adapalene (without niosomes) were prepared using the same procedure [25, 26].

### 2.9. pH and Viscosity Measurement of Ada‐Nio Hydrogel

The pH of the hydrogel was measured using a Sartorius PB‐10 Basic Benchtop pH Meter (Sartorius Co., Germany), and viscosity was determined using a Brookfield DV‐III Ultra digital viscometer (R.V. model, Brookfield, United States). All measurements were performed in triplicate.

### 2.10. In Vitro Release Behavior of Ada‐Nio Hydrogel

The dialysis membrane technique was used to evaluate the release of adapalene from the hydrogel [27]. Ten grams of Ada‐Nio hydrogel and blank hydrogel was placed in separate dialysis bags (molecular weight cutoff = 12,000 Da; Sigma‐Aldrich, Darmstadt, Germany) and immersed in 100 mL of 80% (v/v) methanol:DMF (50:1) in phosphate buffer (pH 5.6). The samples were stirred at 100 rpm and 32°C for 24 h. At predetermined intervals (0.25, 0.5, 1, 2, 4, 8, 12, and 24 h), 2 mL aliquots were withdrawn and replaced with fresh medium. Adapalene concentration was quantified using HPLC with a previously established calibration curve (*R* ^2^ = 0.9991). All experiments were performed in triplicate.

### 2.11. In Vitro Skin Permeation

Skin permeation was assessed using a Franz diffusion cell with a circulating water bath under occlusive conditions at 37 ± 1^°^C [27]. Mouse dorsal skin was excised posteuthanasia (ketamine/xylazine = 8 : 1), shaved, disinfected with 10% (w/v) povidone‐iodine, and trimmed to remove subcutaneous fat. The receptor compartment of the Franz cell was filled with 15 mL of 75% (v/v) methanol:DMF (50:1) in phosphate buffer (pH 5.6). The skin was mounted without air bubbles between compartments.

Three grams of Ada‐Nio hydrogel (1%) or nonniosomal adapalene hydrogel was applied to the donor compartment. The receptor solution was stirred at 150 rpm, and 1 mL samples were withdrawn at 1, 2, 4, 8, 12, and 24 h, replacing each with fresh medium.

After 24 h, the skin was washed to remove excess gel, the stratum corneum was removed by tape‐stripping, and the dermis/epidermis was separated by heating phosphate buffer to 80°C for 3 min. Adapalene was extracted with 5 mL of methanol:DMF (50:1), filtered (0.45 *μ*m), and analyzed by HPLC.

### 2.12. Skin Irritation Test

Skin irritation was assessed in three New Zealand white rabbits (2–3 kg) obtained from the Center for Reproduction, Breeding, and Research of Laboratory Animals, Hamadan University of Medical Sciences, Iran. Animals were housed under 25 ± 2^°^C, 50%–60% humidity, and a 12 h light/dark cycle. All procedures complied with NIH guidelines and were approved by Hamadan University of Medical Sciences (Ethics Code: IR.UMSHA.REC.1398.819).

The Draize test [28] was performed to evaluate irritation. Rabbit dorsal hair was shaved and disinfected with 10% (w/v) povidone‐iodine. The exposed area was divided into four 2 × 2 cm sites. Ada‐Nio hydrogel, nonniosomal adapalene hydrogel, blank hydrogel, and sodium dodecyl sulfate (20%, positive control) were applied separately. Skin reactions (erythema and edema) were scored at 1, 24, 48, and 72 h according to Table 3.

**Table 3:** Scoring system for erythema and edema in skin irritation testing.

**Grade**	**Erythema**	**Edema**
0	No erythema	No edema
1	Very slight erythema	Very slight edema
2	Well‐defined erythema	Slight edema
3	Moderate to severe erythema	Moderate edema
4	Severe erythema	Severe edema

### 2.13. Stability Tests

To analyze the stability of the Ada‐Nio hydrogel, the optimized formulation was stored at 4°C, and its particle size, PdI, zeta potential, and EE% were evaluated using the methods described in the previous sections at 30, 60, and 90 days after formulation preparation. All measurements were performed in triplicate.

### 2.14. Statistical Analysis

The obtained results are reported as mean ± SD, and the data were analyzed using GraphPad Prism V.8 statistical software (La Jolla, California, United States). One‐way ANOVA followed by Tukey’s post hoc test was used to analyze the data. A *p* value of < 0.05 was considered statistically significant.

## 3. Results

### 3.1. Ada‐Nio Preparation Process

According to the section on “Ada‐Nio Preparation,” 15 different experimental formulations (Ada‐Nio‐1 to Ada‐Nio‐15) were prepared in triplicate, and the optimal formulation was selected based on particle size, PdI, zeta potential, and EE% (Table 4). Statistical analysis using Design‐Expert software identified the best‐fitting model to describe variations in particle size, zeta potential, and EE%.

**Table 4:** Particle size, PdI, zeta potential, and EE% of the Ada‐Nio formulations.

**Run**	**Particle size (nm)**	**Zeta potential (mV)**	**PDI**	**EE%**
Ada‐Nio‐1	304.2	80.4	0.79	63.59
Ada‐Nio‐2	186.7	55.1	0.566	86.42
Ada‐Nio‐3	293.1	78.6	0.4	71.1
Ada‐Nio‐4	260.9	−44.4	0.457	43.06
Ada‐Nio‐5	220.1	−49.2	0.434	55.67
Ada‐Nio‐6	295.1	−44	0.378	81.12
Ada‐Nio‐7	204.1	40.1	0.363	60.3
Ada‐Nio‐8	205	65.6	0.549	62.26
Ada‐Nio‐9	231.8	76.7	0.56	84.75
Ada‐Nio‐10	190.1	53.4	0.371	60.2
Ada‐Nio‐11	181.4	60.9	0.304	59
Ada‐Nio‐12	168.1	72.5	0.428	74.2
Ada‐Nio‐13	181.6	60.1	0.488	81.28
Ada‐Nio‐14	299.7	47.8	0.726	80.3
Ada‐Nio‐15	230	−50.1	0.396	44.21

*Note:* The results were expressed as mean ± SD (*n* = 3).

### 3.2. Particle Size

The nanoparticle sizes of the Ada‐Nio formulations ranged from 168 to 304 nm (Table 4). Table 5 summarizes the significant effects of the independent factors on particle size. The lack‐of‐fit test measures a model’s inability to represent laboratory data that are not included in the regression. If the *p* value was greater than 0.05, the model was considered to fit the laboratory data well.

**Table 5:** Effects of independent factors on the particle size.

**Source**	**F** **-value**	**p** **value**	
*Model*	7.19	0.0092	Significant
(C) S:C ratio	8.52	0.0224	Significant
*A* *B*	9.48	0.0178	Significant
*A* *C*	6.55	0.0376	Significant
*B* *C*	10.61	0.0139	Significant
*B* ^2^	12.23	0.0100	Significant
*Lack of fit*	4.08	0.2083	Not significant

The parameters related to the proposed model for changes in nanoparticle size are listed in Table 6. According to Table 6, the *R*‐squared and adjusted *R*‐squared values are nearly equal, and the adequate precision exceeds 4.00. These findings indicate the high predictive power of the model.

**Table 6:** Parameters related to changes in particle size of nanoparticles.

**R** **-square**	**Adj** **R** **-square**	**Pred** **R** **-square**	**Adeq precision**
0.8780	0.7559	0.2275	8.3916

The statistical analysis performed using Design‐Expert 7.0.0 software identified the best‐fitting and statistically significant model to describe particle size variations. A stepwise method was used to remove nonsignificant factors and improve the model’s predictability. Regression analysis showed that the coefficients for factors *B* and *C* were significant. The following equation provides the coefficients of the significant variables affecting particle size:

(1) Y1207.086:+−14.5375∗A+−1.1125∗B+24.7∗C+36.85∗AB+−30.625∗AC+38.975∗BC+43.32682∗B.

Here,

*Y*_1_: nanoparticle size

*A*: adapalene (milligram)

*B*: CTAB (milligram)

*C*: Span 60/cholesterol ratio

**Figure 1:**
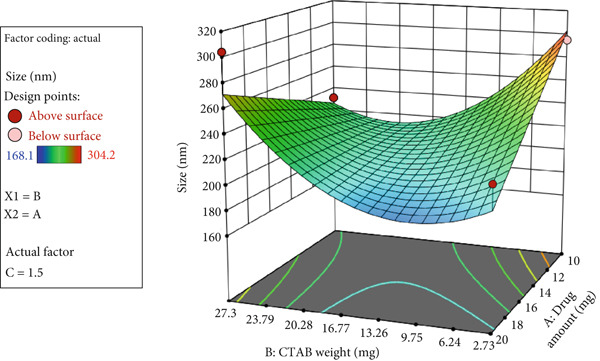
3D response surface plot showing nanoparticle size as a function of Ada amount, Span 60‐to‐cholesterol ratio, and CTAB concentration.

A three‐dimensional (3D) response surface plot of particle size is shown in Figure 1. As illustrated, reducing the Span 60‐to‐cholesterol ratio and increasing the amount of CTAB decreased the size of Ada nanoparticles. However, the amount of Ada did not significantly affect nanoparticle size.

### 3.3. Zeta Potential

The zeta potentials of the Ada‐Nio formulations ranged from −44 to +80 mV (Table 4). Both the CTAB concentration and its quadratic term (CTAB^2^) had a significant effect on nanoparticle zeta potential (*p* < 0.05) (Table 7).

**Table 7:** Effects of independent factors on the zeta potential.

**Source**	**F** **-value**	**p** **value**	
*Model*	427.62	< 0.0001	Significant
*B-CTAB*	718.12	< 0.0001	Significant
*B* ^2^	137.13	< 0.0001	Significant
*Lack of fit*	0.0789	0.9982	Not significant

The parameters related to the proposed model for zeta potential variation are listed in Table 8. These values demonstrate the high predictive power of the model.

**Table 8:** Parameters describing changes in nanoparticle zeta potential.

**R** **-square**	**Adj** **R** **-square**	**Pred** **R** **-square**	**Adeq precision**
0.9862	0.9839	0.9804	42.3711

Regression analysis showed that factors *B* and *B* ^2^ were statistically significant. The following equation provides the coefficients of the significant variables affecting zeta potential:

(2) Y254.71:+61.99∗B+−39.652∗B.

Here,

*Y*_2_: zeta potential

*B*: CTAB (milligram)

**Figure 2:**
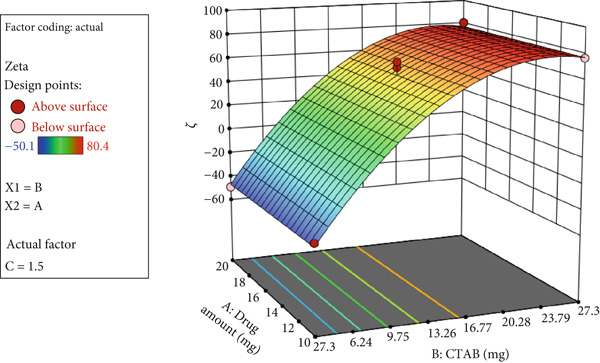
3D response surface plot showing nanoparticle zeta potential as a function of Ada amount and CTAB concentration.

A 3D response surface plot of zeta potential is shown in Figure 2. As illustrated, increasing CTAB concentration raised the zeta potential of nanoparticles, whereas Ada amount did not significantly affect particle size.

### 3.4. EE%

The EE% of the Ada‐Nio formulations ranged from 43.6% to 86.42% (Table 4). Both Ada concentration and CTAB concentration significantly influenced EE%, whereas the Span 60‐to‐cholesterol ratio had no significant effect (Table 9).

**Table 9:** Effects of independent factors on the EE (%).

**Source**	**F** **-value**	**p** **value**	
*Model*	9.12	0.0025	Significant
*A* *-Drug amount*	12.80	0.0059	Significant
*B* *-CTAB*	12.01	0.0071	Significant
*A* ^2^	16.68	0.0027	Significant
*Lack of fit*	23.77	0.0409	Significant

The parameters related to the proposed model for EE% variation are listed in Table 10. These results confirm the strong predictive ability of the model.

**Table 10:** Parameters describing changes in the EE% of nanoparticles.

**R** **-square**	**Adj** **R** **-square**	**Pred** **R** **-square**	**Adeq precision**
0.8351	0.7435	0.4418	9.5785

Regression analysis showed that factors *A*, *B*, *C*, AC, and *A* ^2^ were statistically significant. The coefficients of these variables affecting EE% are provided in the following equation:

(3) Y359.16:+−8.98∗A+8.7∗B+1.63∗C+6.8∗AC+15.002∗A.

Here,

*Y*_3_: EE (%)

*A*: adapalene (mg)

*B*: CTAB (milligram)

*C*: Span 60/cholesterol ratio

**Figure 3:**
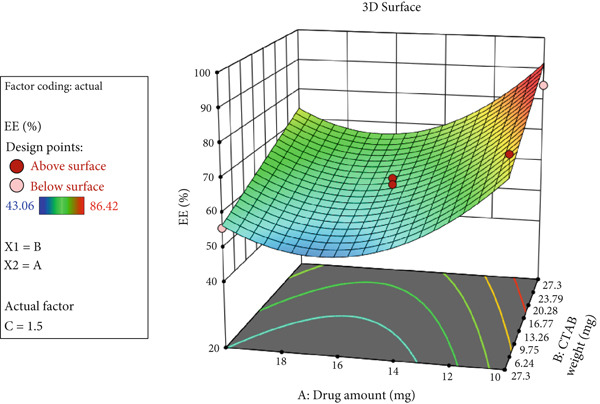
3D response surface plot showing changes in nanoparticle EE% based on variations in Ada amount and CTAB concentration.

The 3D response surface plot of EE% is shown in Figure 3. As illustrated, EE% increased as the amount of CTAB increased, but decreased as the amount of Ada decreased.

### 3.5. Model Optimization and Validation

The statistical analysis performed using Design‐Expert software was applied to select the most statistically significant model to describe changes in particle size, zeta potential, and EE (%). Table 11 presents the predicted conditions for preparing the optimal nanoparticles. Accordingly, the optimal formulation was prepared with 10.921 mg of Ada, 64.2 mg of Span 60, 61.2 mg of cholesterol, and 24.6 mg of CTAB. The probability of achieving these predicted conditions was 0.99.

**Table 11:** Optimized independent variables and predicted responses.

**Optimized independent variables (factors)**			**Predicted dependent variables (responses)**			**Desirability**
**Drug amount (mg)**	**Span 60/cholesterol ratio**	**CTAB amount (mg)**	**Size (nm)**	**EE (%)**	**Zeta potential**	
10.921	1.049	24.624	148.788	86.816	78.940	0.996

**Figure 4:**
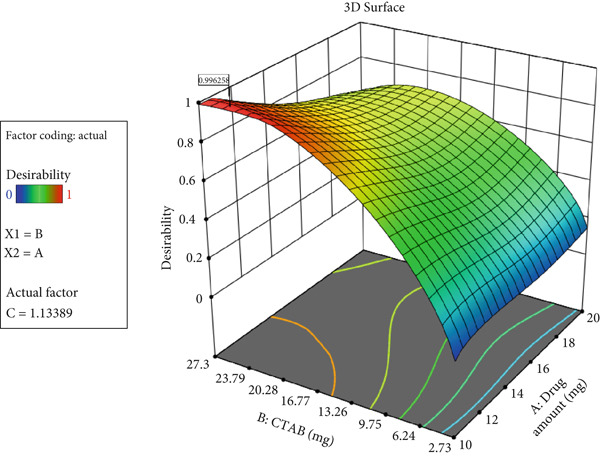
Probability of achieving predicted conditions based on variations in drug amount, Span 60‐to‐cholesterol ratio, and CTAB concentration.

As shown in Figure 4, using this formulation provides the highest probability of obtaining optimal nanoparticles.

To validate the model and determine the prediction error, nanoparticles were prepared in triplicate in the laboratory according to the optimal conditions suggested by the model, and their characteristics were evaluated. As shown in Table 12, the prediction error percentage was less than 25%, indicating the models are significant, accurate, and predictive.

**Table 12:** Observed responses for the predicted optimized formulation (*n* = 3).

**Experimental (observed)**		**Prediction error (%)**
**Size (nm)**		11.9
Observed response (mean ± SD)	168.8	
**EE%**		8.1
Observed response (mean ± SD)	80.3	
**Zeta potential (mV)**		11.8
Observed response (mean ± SD)	70.6	

### 3.6. Ada‐Nio Morphology

**Figure 5:**
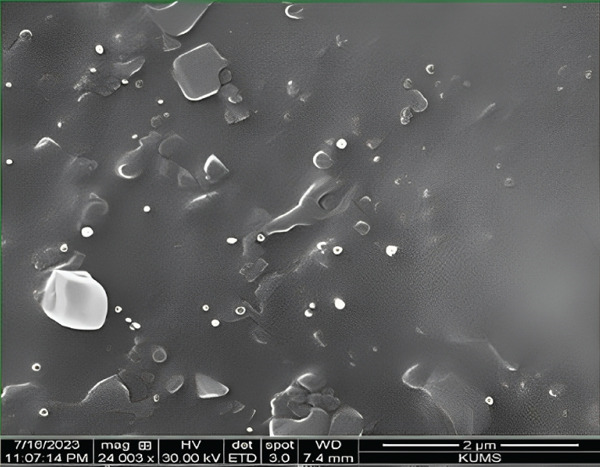
SEM image of optimized Ada‐Nio nanoparticles.

As shown in Figure 5, the size of the optimized Ada‐Nio nanoparticles corresponds with previous results and exhibits spherical or oval shapes without aggregation.

### 3.7. Cytotoxicity

**Figure 6:**
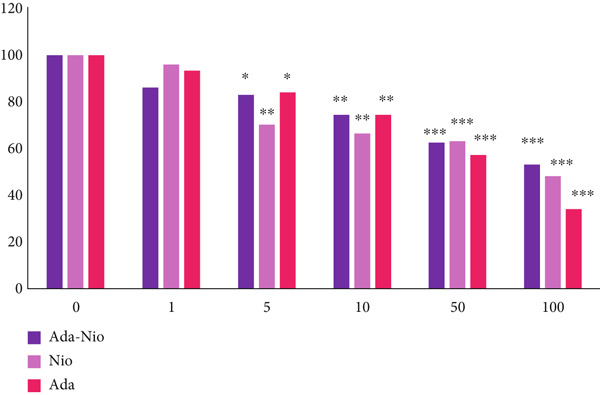
HDF cell viability assay in different concentrations of adapalene (Ada), Ada‐Nio, and blank noisome (Nio). The obtained results were reported as mean ± SD (*n* = 3).  ^∗^ *p* < 0.05,  ^∗∗^ *p* < 0.01, and^∗∗∗^ *p* < 0.001 as compared to the control group.

The cytotoxicity of adapalene, Ada‐Nio, and blank noisome on HDF cells using the MTT assay after 48 h is shown in Figure 6. Cell viability at concentrations higher than 1 *μ*g/mL was < 80% after 48 h. At concentrations higher than 10 *μ*g/mL, cell viability in all groups fell below 70%, indicating that adapalene exhibits cytotoxicity toward HDF cells. Based on these results, a concentration of 1 *μ*g/mL was selected as a safe dose for further studies.

### 3.8. pH, Viscosity, and Appearance of Ada‐Nio Hydrogel

The pH, viscosity, homogeneity, and color of 0.1% Ada‐Nio hydrogel were evaluated, and results are shown in Table 13. The pH and viscosity were acceptable at 6.4 and 33,840 cP, respectively.

**Table 13:** pH, viscosity, homogeneity, and color of 0.1% Ada‐Nio hydrogel. All results are expressed as mean ± SD (*n* = 3).

**Formulation**	**pH**	**Viscosity (cP)**	**Homogeneity**	**Color**
Ada‐Nio hydrogel 0.1%	6.4 ± 0.3	33,840 ± 1200	Acceptable	White

### 3.9. In Vitro Release Studies

**Figure 7:**
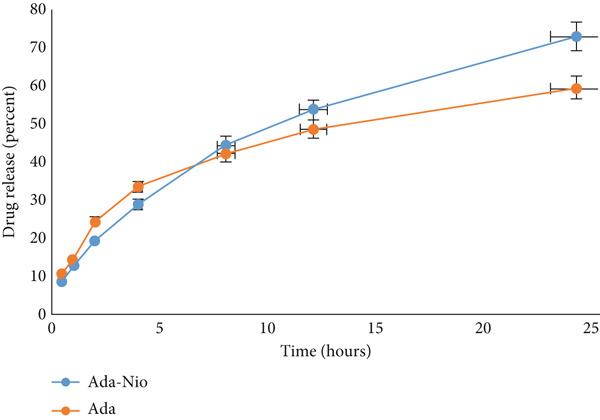
In vitro release of adapalene (Ada) and Ada‐loaded niosomal hydrogel (Ada‐Nio) over 24 h.

The in vitro drug release of 0.1% Ada‐Nio hydrogel was evaluated. Sampling was stopped after 24 h for two reasons: [1] Drug release reached a stable plateau, and [2] for local dermal application, there is no need to assess release beyond 24 h. Figure 7 shows the release profiles of free adapalene and Ada from the niosomal hydrogel. Drug release was rapid during the first 4 h, likely due to unentrapped Ada on the nanoparticle surface. Adapalene was then gradually released from the hydrogel over 24 h, with approximately 73% released, compared to 58% release for free Ada.

### 3.10. In Vitro Permeation Studies

**Figure 8:**
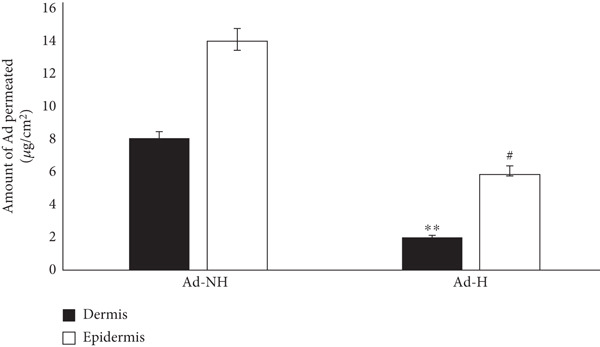
Comparative in vitro permeation study for Ada‐Nio hydrogel and Ada hydrogel using Franz cell. Data are mean ± SD (*n* = 3).  ^∗∗^ *p* < 0.01 as compared dermis, #*p* < 0.01 as compared epidermis.

The drug content in the epidermis and dermis layers of mouse skin is shown in Figure 8. The Ada‐Nio hydrogel delivered a significantly higher amount of adapalene to both dermis and epidermis layers compared to Ada hydrogel. In contrast, the in vitro permeation of Ada hydrogel was significantly lower than Ada‐Nio hydrogel in the dermis (*p* < 0.01) and epidermis (*p* < 0.05).

### 3.11. Skin Irritation Studies

Based on visual observations of erythema and edema severity at 1, 2, 24, 48, and 72 h, skin reaction results are summarized in Table 14. Results indicated that although Ada hydrogel caused mild erythema at 1 and 2 h, Ada‐Nio hydrogel caused no visible irritation, suggesting that it can be considered a safe topical formulation.

**Table 14:** Evaluation of skin irritation using the Draize test.

**Hours**	**Groups**							
	**SDS**		**HB**		**Ada-H**		**Ada-Nio-H**	
	**Erythema**	**Edema**	**Erythema**	**Edema**	**Erythema**	**Edema**	**Erythema**	**Edema**
1	2	0	0	0	2	0	0	0
2	3	1	0	0	1	0	0	0
24	1	0	0	0	0	0	0	0
48	1	0	0	0	0	0	0	0
72	1	0	0	0	0	0	0	0

Abbreviations: Ada‐H, adapalene‐loaded hydrogel; Ada‐Nio‐H, adapalene‐loaded niosomal hydrogel; HB, blank hydrogel group; SDS, sodium dodecyl sulfate.

### 3.12. Stability Tests

The particle size, PDI, zeta potential, and EE% of Ada‐Nio hydrogel were evaluated at 30, 60, and 90 days, and the results are summarized in Table 15. The particle size, PDI, zeta potential, and EE% remained within acceptable ranges even after 90 days, indicating good stability of the formulation.

**Table 15:** Stability parameters of Ada‐Nio hydrogel at 30, 60, and 90 days. All values are expressed as mean ± SD (*n* = 3).

**Day of storage**	**Particle size (nm)**	**PdI**	**ZP (mV)**	**EE (%)**
0	168	0.2	70.6	80
30	193	0.3	63.2	68
60	234	0.5	65.7	63
90	297	0.6	62	59

## 4. Discussion

In the current study, a Box–Behnken (BB) design was employed to statistically optimize the variables of the topical adapalene‐loaded niosomal formulation. The optimized niosomes exhibited small particle sizes, good stability, and high entrapment efficiencies. In vitro skin deposition studies indicated that niosomes enhanced drug permeation into the skin compared to the drug solution. These findings suggest that Ada‐Nio could have significant therapeutic applications in the topical treatment of acne. However, further preclinical studies using appropriate animal models are necessary to confirm the therapeutic efficacy and safety of the developed niosomes [29].

The optimized nanoparticle’s size, PDI, zeta potential, and EE% of Ada‐Nio formulations were 168.8 ± 1.5 nm, 0.273 ± 0.015, 70.6 ± 1.05 mV, and 80.3 ± 2.8*%*, respectively. The various formulations demonstrated that reducing the ratio of surfactant (Span 60) to cholesterol, while increasing the amount of CTAB, led to a decrease in nanoparticle size. Additionally, the quantity of the drug did not significantly impact the size of the nanoparticles. Particle size plays a crucial role in drug permeation, biodistribution, and delivery efficiency [30].

In addition, PDI refers to the uniformity of particle size distribution, and a value less than 0.5 is considered indicative of a monodisperse nanoformulation [31]. Furthermore, by increasing the amount of CTAB, the zeta potential of nanoparticles increased. Zeta potential is an important parameter for assessing nanoparticle colloidal stability. This parameter indicates the surface charge of the nanoparticles and helps prevent coalescence or aggregation, contributing to long‐term colloidal stability [32]. CTAB is a cationic surfactant that can increase colloidal stability [33]. However, the highest zeta potential among the 15 different formulations corresponded to the Ada‐Nio formulation with the highest amount of CTAB (27.3 mg). Large negative or positive zeta potential values indicate satisfactory colloidal stability.

EE% is an important factor for the effectiveness of nanoformulations and reflects the percentage of drug incorporated into carriers [34]. By reducing the amount of cholesterol and increasing the amount of Span 60, the percentage of nanoparticle encapsulation increased. It has been reported that cholesterol decreases EE% because it disrupts the normal bilayer arrangement [35].

Shah et al., in a 32 fractional design, reported that the particle size, PDI, zeta potential, and EE% of the optimized Ada‐Nio batch were 278 nm, 0.727, −17.99 mV, and 86.07%, respectively. In addition, their findings revealed that intermediate values of Span 60 and cholesterol decreased particle size and increased zeta potential, which align with our findings [36].

SEM facilitates the characterization of solid nanoparticles. The SEM image of Ada‐Nio showed approximately spherical nanoparticles, which are consistent with their unique structural features. Several studies have reported similar spherical shapes for drug‐loaded niosomes, consistent with the Ada‐Nio morphology [11, 37].

The cytotoxicity of adapalene, Ada‐Nio, and blank noisome was evaluated using the MTT assay in HDF cell lines. The results revealed that concentrations below 1 *μ*g/mL were nontoxic, whereas higher concentrations showed a positive correlation with cytotoxicity. Therefore, 1 *μ*g/mL was selected as the safe dose for further studies. Notably, Ada‐Nio exhibited higher cell viability than the blank noisome, consistent with previous reports that drug‐loaded niosomes can reduce toxicity while increasing therapeutic effectiveness [38, 39].

Ada‐Nio hydrogel was satisfactorily prepared and showed a pH of 6.4, a viscosity of 33,840 cP, white color, and uniform homogeneity. A pH range of 5.5–7 is appropriate for dermal absorption, as the skin surface has a pH of approximately 5.5, which rises to 7 in the inner skin strata [40]. Arooj et al. reported that adapalene‐loaded liposomal hydrogel had a pH of 6.43 [41].

Moreover, the viscosity of Ada‐Nio hydrogel was acceptable. For instance, Pawar et al. reported a viscosity of 29,548 cP for adapalene‐loaded solid lipid nanoparticle hydrogel [42]. Higher viscosity enhances skin penetration and prolongs the contact time between the drug and skin, improving diffusion [43, 44].

The in vitro release behavior of adapalene from the hydrogel reached approximately 73% in a sustained profile over 24 h. The initial burst release could be related to unentrapped adapalene on the surface of the nanoparticles, while sustained release may result from slow diffusion from the hydrogel matrix. Encapsulation in niosomes controls Ada release, indicating that the integrity of niosomes is maintained within the hydrogel [44]. Similar studies by Vasanth et al. [45] and Shilakari et al. [46] reported a gel matrix surrounding niosomes, achieving controlled drug release and improved skin absorption.

In vitro permeation studies compared Ada‐Nio hydrogel and Ada hydrogel using Franz cells. Ada‐Nio hydrogel showed significantly higher diffusion into the dermis and epidermis layers than Ada hydrogel. Drug absorption from the stratum corneum occurs via transepidermal and transappendageal pathways [47]. The transepidermal route includes transcellular and intercellular pathways, in which hydrophobic drugs diffuse through SC cells composed of lipid bilayers [48] and are then transported to dermal capillaries [49]. The lipophilic nature of Ada and the niosome composition may enhance SC permeation, consistent with Arooj et al. [29]. Moreover, transdermal delivery bypasses first‐pass metabolism, further improving drug penetration [50].

Skin irritation studies revealed that Ada‐Nio hydrogel is safe and nonirritant, suitable for controlled topical delivery. Mechanistically, Ada has a high affinity for retinoic acid receptor‐*γ* (RAR‐*γ*) and RAR‐*β* in the epidermis and dermal fibroblasts, inhibiting keratinocyte differentiation [51]. Although erythema, dryness, and burning are common Ada side effects, 0.1% Ada cream exhibits better tolerability and acceptance [52, 53]. Various formulations, including liposomal gels [54], nanostructured lipid carriers [55], microsphere gel formulations [56], microemulsions [57], and niosomal gels [54], have been explored. Consistent with Shah et al., Ada‐Nio hydrogel showed a lower primary irritation index (PPI), with no signs of skin irritation.

This study has two main limitations. First, the availability and cost of materials and the lack of some laboratory equipment required collaboration with other laboratories. Second, the limited solubility of adapalene restricted the study to specific solvents and compounds validated in similar studies.

Furthermore, these findings suggest that future studies could explore the synergistic effects of other antiacne drugs combined with adapalene in niosomes to create multifunctional products and enhance patient compliance.

## 5. Conclusion

In this study, Ada‐Nio was successfully prepared in nanosized form with high EE%. The evaluation of different formulations revealed that the amounts of Span 60, cholesterol, and CTAB significantly influenced particle size, zeta potential, and EE%. Ada‐Nio at 1 *μ*g/mL was found to be noncytotoxic to HDF cell lines. Based on the optimized formulation, Ada‐Nio hydrogel was prepared, exhibiting acceptable pH, viscosity, and homogeneity. The hydrogel demonstrated spherical nanoparticles, sustained drug release, and high in vitro skin permeation. Importantly, no signs of skin irritation were observed in rabbit skin studies. Overall, this study highlights the therapeutic potential of Ada‐loaded niosomal gel, suggesting that Ada‐Nio hydrogel represents a promising platform for novel topical formulations in acne therapy.

## Conflicts of Interest

The authors declare no conflicts of interest.

## Funding

This work was supported by the Deputy of Research and Technology, Hamadan University of Medical Sciences, Hamadan, Iran, under grant number 9811088688.

## Data Availability

The data supporting the findings of this study are available from the corresponding author upon reasonable request.
